# The oral case presentation: toward a performance-based rhetorical model for teaching and learning

**DOI:** 10.3402/meo.v20.28565

**Published:** 2015-07-17

**Authors:** Mei Yuit Chan

**Affiliations:** Faculty of Modern Languages and Communication, Universiti Putra Malaysia, Serdang, Selangor, Malaysia

**Keywords:** oral case presentation, medical case presentation, performance-based model, rhetorical model, rhetorical analysis, expert presentations, intertextual analysis, genre analysis, medical education, communication skills

## Abstract

The oral case presentation is an important communicative activity in the teaching and assessment of students. Despite its importance, not much attention has been paid to providing support for teachers to teach this difficult task to medical students who are novices to this form of communication. As a formalized piece of talk that takes a regularized form and used for a specific communicative goal, the case presentation is regarded as a rhetorical activity and awareness of its rhetorical and linguistic characteristics should be given due consideration in teaching. This paper reviews practitioners’ and the limited research literature that relates to expectations of medical educators about what makes a good case presentation, and explains the rhetorical aspect of the activity. It is found there is currently a lack of a comprehensive model of the case presentation that projects the rhetorical and linguistic skills needed to produce and deliver a good presentation. Attempts to describe the structure of the case presentation have used predominantly opinion-based methodologies. In this paper, I argue for a performance-based model that would not only allow a description of the rhetorical structure of the oral case presentation, but also enable a systematic examination of the tacit genre knowledge that differentiates the expert from the novice. Such a model will be a useful resource for medical educators to provide more structured feedback and teaching support to medical students in learning this important genre.

The oral case presentation (OCP) is a key genre in medical communication among medical practitioners. It is also a means by which medical students are evaluated for their competence in medical knowledge and clinical reasoning skills. Failure to acquire the skills required for an effective case presentation by medical students will result in much frustration among students and their teachers, given the central role of the OCP in medical practice and education. However, despite the importance of the OCP, teaching and learning support provided to students to help them acquire this form of communication has been found to be lacking.

Students’ inadequate mastery of the OCP has been a concern among medical lecturers and language and communication lecturers (1–5). Medical lecturers are quick to recognize OCPs presented by students as unsatisfactory, but the feedback provided to students oftentimes could not adequately convey the finer requirements of the OCP (2). Simplistic ‘rules’ on how to present effectively provided in pre-clinical briefings, and lecturers’ feedback to students during clinical teaching sessions sometimes serve to confuse more than clarify what students have done wrong and how they should repair their presentations (4).

## The case presentation as a rhetorical and linguistic activity

A main reason why the teaching of the OCP poses a challenge to lecturers is because of the complex rhetorical nature of the activity (4, 5). Foremost is the fact that the OCP is required to be presented extemporaneously (without reading from a written text) (6) making it a daunting task for novices who have not yet mastered the art of thinking, composing, and talking on their feet. Also, this type of talk has to take into account audience, purpose, time, and urgency (4, 7, 8), making it a clear rhetorical act.

It is a misrepresentation to assume that scientific content or medical knowledge and clinical reasoning can be conveyed or demonstrated effectively without rhetorical skills and an understanding of how language works. Dell et al. (1) in presenting a guide for good presentations and the pitfalls to avoid, reaffirm the interrelation between effective OCPs and clinical reasoning. Medical lecturers and practitioners have noted the importance of rhetorical and linguistic skills (although not using these terms specifically) in their advice to students on how to present cases effectively. For example, Green (2) points out that telling a good ‘story’ which includes ‘weav(ing) facts from the history of present illness (HPI) into a coherent narrative that summarizes the events that led up to the patient presentation’, proper organization, a convincing argument, pertinent or relevant information, and speaking fluency, are necessary in a good presentation. Bushan et al. (9) note the importance of delivery skills, stating that ‘a great presentation requires style as much as substance; your delivery must be succinct and smooth’, and ‘it is not simply a regurgitation (of the written report)’. In the University of Washington's website (6) offering information to help students deliver a good OCP, students are given advice on what content to be conveyed for each section of the OCP, and tips for effective delivery which include ‘keep your language precise’, ‘use positive statements rather than negative statements’, and ‘do not rationalize or editorialize as you present’. Colgan (10) reaffirms the interconnection between content and the manner it is presented when he observes that ‘students, residents, and even physicians present in a haphazard way. These people often have intelligent contributions to make to the discussion, but their message is often lost in the confusion and disarray of ideas’.

Apart from practitioners providing guidance to students on how to present the OCP drawn from their experience, there are, too, a few published research studies that have attempted to shed light on the characteristics of the OCP and how it can be best taught and assessed. Green et al. (3) found that the attributes of the OCP regarded to be important to internal medicine faculty from five medical schools in the U.S. have among them, numerous characteristics that possess rhetorical and linguistic significance, such as ‘accurate description of the symptoms’, ‘organized according to usual standards’, ‘reports sequence of events that preceded the current hospitalization’, ‘structured to guide the listener to the same conclusions as the speaker (e.g., makes a case)’.

In another initiative of a similar nature, Lewin et al. (11, 12) developed a rating scale containing various aspects of the OCP. The rating scale contains such specifications that are directly or indirectly referenced to organizational, rhetorical, and linguistic ability in performing the OCP. For example, the specification ‘Chief complaint noted either before HPI or as part of introductory sentence’ (12) presupposes awareness of a sentence structured to function as an introductory sentence and that should contain certain required information. The specification ‘HPI is organized so that chronology of important events is clear’ expects the student to be able to utilize rhetorical and linguistic strategies and devices to index events as important, and mark the time sequence of their occurrence. Interestingly, under the section ‘General aspects’, there are two categories which are ‘Overall organization’ and ‘Speaking style’, with the latter having the rubrics that describe whether the presentation is ‘easy to understand’ and whether an ‘engaging speaking style’ is demonstrated. While all good communication is expected to be coherent and comprehensible, the inclusion of an interesting and interactive speaking style that characterizes the term ‘engaging’ without a doubt marks the important rhetorical nature of the OCP.

Dell et al. (1), in their opinion article, listed the skills required for students to produce a high quality presentation. As in the two previous studies cited, a large proportion of the descriptors provided have important rhetorical and linguistic implications. A selection of relevant examples are ‘summarize case by using descriptive adjectives to describe key features’, and ‘educate colleagues through presentations’ (1). Just as the description of a good presentation is informative about its rhetorical requirements, the description of a bad presentation can provide insight into rhetorical skills ‘misapplied’. A poor presentation is indicated with descriptors such as ‘disorganization’, ‘exhaustive report of irrelevant details’, ‘case summary only repeats factual details’, ‘no plan discussed, or plans offered as random “to do” list’, and ‘cannot explain plan to others’ (1).

All of the characteristics of the OCP reviewed above are descriptions about what content should be included, and more importantly, how this content should be conveyed. For example, indicator words such as summarizing, discussing, describing, noting, stating, conveying (10), educating (1), weaving facts (2), and making a case (3), all represent different rhetorical acts. Further, these rhetorical acts occupy a place in a hierarchical system where larger scope rhetorical acts are realized by a series of smaller (subordinate) speech acts, which in turn, are realized by specific linguistic structures and lexical selections. Acts such as summarizing, and educating or making a case are clearly placed at a higher level in the hierarchy compared to noting, listing, and stating.

At the level of linguistic choice, with the addition of the adverbial ‘accurately’ to the act ‘describing symptoms’ in Green et al.'s description of a good presentation, for example (3), one might expect the student (presenter) to use and apply appropriate lexical resources to describe the quality of the symptoms reported by patients, and possibly use appropriate intensifying or mitigating devices to convey accurately the extent of the symptoms. Similarly, Dell et al.'s inclusion of the additional condition specified in the qualifying phrase ‘by using descriptive adjectives’ (1) within the act of summarizing a case shows the authors’ awareness of the important role of language as the mediator of meaning in communication. Here, students are expected to use appropriate words in carrying out the rhetorical action specified.

It is not unusual for a student who is instructed to ‘discuss’ or ‘make a case’ to wonder how it should be carried out. The lack of a clear description of what lower level acts should be performed in order to accomplish the higher level acts results in many students learning the OCP through observation and trial and error. Hence, teachers paying more attention to the rhetorical aspect of the OCP would be a step in the right direction in helping students to acquire this form of communication.

## The need for a description of genre and the underlying genre knowledge

While the importance of teaching the OCP to students is not disputed, how one should go about teaching it is less clear. The lack of a rhetorical model of the OCP may be a contributing factor to why students report learning the OCP in an ad-hoc manner (4). There is presently no systematic description of the structure, language, and function of the OCP from the linguistic standpoint. What is currently available representing the macrostructure of the OCP comprises the topical categories known to all medical practitioners as the basic structure, namely the ‘chief complaint’, ‘history of present illness’, ‘past history’, ‘family history’, ‘social history’, ‘physical examination’, ‘diagnostic impressions’, and ‘management plan’ (13), with slight variations in the labeling of the categories used by different medical schools. While practical guidelines, suggestions, feedback, and tips from medical lecturers on how to make a good case presentation are often given to students, the performance of students has been lamented to be mostly unsatisfactory, and students likewise have reported confusion and frustration about their own ability to produce the OCP (2, 13). This is not surprising, as the OCP is not merely a structured piece of text type whose surface patterns can be acquired as a set of rules. Lingard et al. (8, 14) found that students regard the OCP as an inflexible template that should be followed strictly, whereas doctors expect that content and structural elements of the OCP should be modified to suit the case, situation, and audience, but at the same time, the basic structure is adhered to. Undeniably, there is much in the way of expert knowledge that must be brought to bear in the process of composing the OCP, that manifests in the final piece of spoken text that differentiates the expert status of the doctor from the novice position of the student.

## Building a model from expert opinion

Previous studies have tried to synthesize this expert knowledge that is mainly tacit in nature into an explicit description, so as to make the teaching and learning of the OCP more accessible. Most of these studies have utilized the methodological approach of drawing on expert opinion; that is, the gathering of opinions from medical lecturers and practitioners about what they thought constitute effective or ineffective presentations (3, 11, 12), obtained either through direct communication with expert subjects, or through observation of feedback given by medical lecturers to students about their performance in teaching sessions (4, 8, 13, 14). While interviewing experts has much value in obtaining snippets of insight into the expectations of the expert discourse community concerning how an OCP should be delivered, such methods of depicting expert knowledge have their limitations. As asserted by Sarangi (15) in explaining the difficulty of researching professional competencies, ‘a profession's knowledge base operates mainly at a tacit level’, and citing Schon (16) who noted that ‘competent practitioners usually know more than they can say’. Furthermore, the use of language itself, a large part of which is procedural knowledge in human communication, is a predominantly automatic process that operates at the subconscious level (17). It is difficult for an individual to describe accurately at the microlevel the linguistic and rhetorical knowledge and skills that he or she draws on and what motivation he or she is driven by when performing a communicative act, from retrospective memory. This ‘knowledge-in-action’ or tacit knowledge is the knowledge that experts use when performing professional tasks, but are not able to articulate about in sufficient detail (16).

Observation of student presentations and lecturers’ situated feedback to these presentations provide another useful avenue to getting at what lecturers expect in a good presentation. However, such feedback is often unstructured, and arises in bits and pieces depending on the focus of the lesson, type of errors made by students, time constraints, personality of the lecturer, and other situational variables.

## Toward a performance-based model

To obtain a more comprehensive model of the OCP that would be useful to teachers and students, expert opinion derived from interviews and observations should be supplemented with the analysis of expert performance, that is, the analysis of actual presentations produced by the expert members of the community themselves. This calls for a shift from relying solely on models derived from expert opinion about effective and ineffective presentations toward a model based on the close analysis of experts’ actual production of the communication type in question. There are many advantages in taking the approach of deriving a model from analyzing a set of pooled actual presentations by experts. Foremost is the objectivity by which claims can be made by the analyst about experts' actual patterns of use of language and rhetorical resources, which are demonstrated in their presentations. In this regard, the stable set of recorded talk data allows for inter-rater consistencies in the analysis and hence, enables more reliable and valid claims. Another important advantage is that a model built specifically for the purpose of training new members should ideally be developed based on input from the expert members of the community who would be teachers, mentors or evaluators of the newcomers. Hence, a locally situated model derived from within particular institutional, national, or geographic boundaries has its benefits as compared to a more generalized model that aims to represent a prototype for the profession. The following section discusses the theoretical framework that can support this type of initiative.

## Theoretical perspective and analytical tool

The theoretical basis for such a project is derived from discourse and genre theories (18–21) both of which are widely used in applied linguistics research. Discourse is viewed as social action, which can be systematically analyzed through the close examination of text (both written and spoken language). Texts that occur in identifiable regular patterns to perform particular social actions in recurrent social and institutional contexts are known as genres. Genre analysis has contributed to educational enterprises by enabling a systematic description of the linguistic forms and the rhetorical actions served by these forms in various academic and professional genres to support the teaching of these genres to novices. Combining genre analysis with the notion of intertextuality (21–24) that takes into account prior genres along the chain of communication, it is possible to reveal the tacit genre knowledge that guides the production of a subsequent genre.

The importance of including the analysis of intertexts in genre analysis can be understood within the broad vision of genre theory proposed by Bhatia (21) that does not restrict genre analysis to only structural description, but aims to explain how members of a discourse community produce and use genres. When the process of recontextualizing from a salient prior genre to a current one is compared between experts and novices, differences in the patterns of task performance by the experts and the novices will provide invaluable insight into the tacit genre knowledge experts draw on when producing the genre of interest to the study. Hence, genre analysis is not merely descriptive, but its usefulness extends toward explanation; it is able to explain the production, manipulation, consumption, or transformation of genres by members of the discourse community, to answer questions that have social and educational significance.

**Fig. 1:**
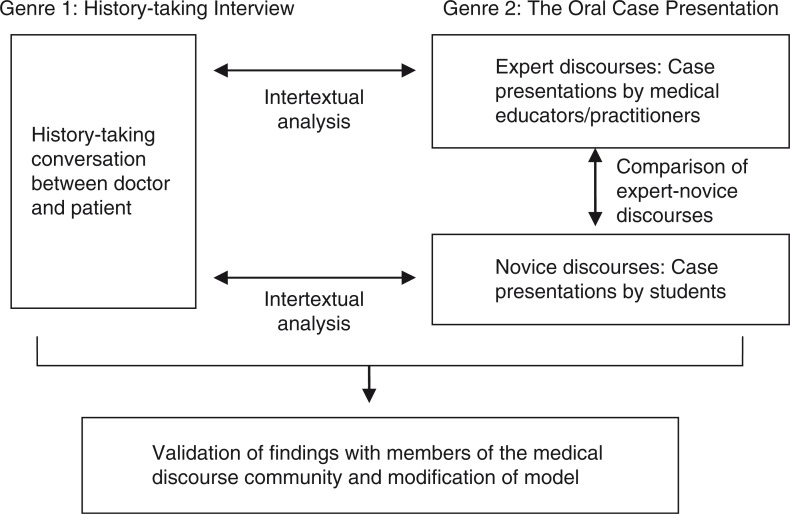
Application of genre and intertextual analysis in researching expert-novice performance and tacit genre knowledge.

In the context of the medical case presentation, the salient genre prior to it is the history-taking activity more commonly known as the medical interview in consultations. The contrast between the two genres of the history-taking interview and the OCP is sharp - one is a dyadic semi-formal conversation and the other a formal monologue report. In order to compose the OCP (current genre), the speaker has to negotiate the intertextual connection between it and its immediate prior genre, the history-taking interview. Figure 1 shows how the genre analytical approach can be applied in developing a performance-based model of the OCP.

## Conclusion

The significance of building a model based on expert–novice performance is that it will be a model anchored in the discourse community from which the model is derived and in which it will be used. Such a model will incorporate and reflect the language patterns, practices, ideology, and culture of the said community. Genre theory and analysis is a powerful tool that would enable not only the systematic description of the rhetorical and linguistic features of a genre, but also the examination of the tacit genre knowledge that members draw on when composing and delivering a text. Development of such a product enabled by interdisciplinary collaboration will serve to enhance the teaching and learning of the OCP. As a final remark, the review and perspective presented in this paper highlight the importance of collaborative effort between medical educators and researchers in the field of linguistics and communication to spur advances in medical educational practices.
